# A Novel Approach to Augmenting Allograft Hamstring Anterior Cruciate Ligament Reconstructions Utilizing a Resorbable Type I Collagen Matrix with Platelet Rich Plasma

**DOI:** 10.1155/2021/5574676

**Published:** 2021-03-10

**Authors:** Sean Mc Millan, Danielle Thorn, Elizabeth Ford

**Affiliations:** ^1^Department of Orthopedics, Virtua Health System, 2103 Burlington-Mount Holly Rd, Burlington, NJ 08016, USA; ^2^Department of Orthopedics, Virtua Health System, Burlington, NJ, USA; ^3^Department of Orthopedics, Inspira Health System, Vineland, NJ, USA

## Abstract

**Background:**

Anterior cruciate ligament reconstruction (ACLR) is one of the most common lower extremity orthopedic surgeries performed in the United States. Annually, between 100,000 and 200,000 ACL tears affect 1 in 3,000 people. The selection of autograft versus allograft for ACLR has been widely discussed in terms of risk of graft failure. Allograft reconstructions have been shown to have higher rerupture rates. One factor contributing to this risk is delayed biologic graft incorporation.

**Methods:**

A retrospective review was performed examining 14 patients who underwent an augmented quadruple-stranded hamstring allograft ACLR with a type I resorbable collagen matrix impregnated with platelet-rich plasma (PRP).

**Results:**

Within our clinical practice, the augmentation of quadruple-stranded hamstring allograft ACLR with a type I resorbable matrix impregnated with PRP has yielded good early clinical success at 2-year outcomes (*N* = 14). Zero ACLR failures have been reported to date in this series.

**Conclusion:**

This case series offers a novel approach for soft tissue allograft ACLR augmented with a type I collagen matrix impregnated with PRP. The authors theorize that this augmentation may improve biologic graft incorporation into the host bone tunnels.

## 1. Introduction

Anterior cruciate ligament reconstruction (ACLR) is one of the most common lower extremity orthopedic surgeries performed in the United States. The anterior cruciate ligament (ACL) is important for the stability of the knee, controlling against excessive motion. Furthermore, the ACL resists anterior tibial translation, while providing rotatory stability and proprioception. Annually, between 100,000 and 200,000 [1, 2], ACL tears affect 1 in 3,000 people in the general population at an estimated cost of $3 billion [3].

ACLR failure can be defined in a multitude of ways. Noyes and Barber-Westin defined ACLR failures requiring surgical correction as: a complete graft tear with >6 mm of anterior tibial displacement as compared to the healthy knee; a positive pivot shift test graded +2 or +3 compared to the healthy knee, with or without knee pain or inflammation, or subjective sensation of instability or functional limitations for daily life and/or sports activities [4]. Factors leading to ACLR failure include surgical technique, failed biology, and rehabilitation protocols.

Poor surgical technique has been identified as a risk of graft failure [5, 6]. More specifically, failure has been associated with mal-positioning of the graft in the femoral tunnel, resulting in increased graft load, abnormal knee kinematics, and/or instability of the knee joint [7]. Similarly, failure of biologic incorporation of the graft been correlated to ACL graft survivorship, specifically in the setting of allograft usage [5–7]. Benefits of autograft tissue for ACLR include decreased risk of tissue rejection, decreased procedural cost, and faster graft incorporation potential. Conversely, allograft tissue is favored in some instances due to decreased donor site morbidity, ease of surgical technique, decreased surgical time, and more predictable graft size [6]. Nevertheless, allograft ACLR has been associated with higher rates of graft failure compared to autograft tissue [8–13].

A primary reason for allograft ACLR failure has been attributed to a lack of biologic incorporation. Achieving biologic incorporation using allograft is a complex process that involves tissue integration, revascularization, and ultimately “ligamentization” into host tissue [14]. Delayed biologic incorporation of allograft tissue compared to autograft tissue has also been noted in the literature [14–17]. Scheffler et al. noted that allograft tissue incorporation was slower at both 6 weeks and 12 weeks compared to the incorporation of autograft tissue. This trend of “incorporation lag” continued out to 52 weeks. Various factors have been theorized as to the delayed incorporation, including allograft processing and purifications methods [14–19].

**Figure 1:**
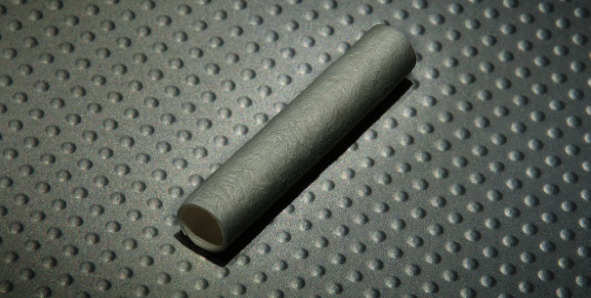
TenoMend™ comes as a machined pre-curled resorbable type I collagen. (Re-Printed with Permission from Exactech, Gainesville, FL).

Consideration of the delicate balance between the attraction of allograft and the respect for biologic graft incorporation is a challenging dilemma for orthopedic surgeons. The introduction of ACLR scaffolds and biologic agents has been discussed to improve healing [20]. A systematic review by Andriolo et al. found a positive impact of platelet-rich plasma (PRP) in ACL graft ligamentization and inflammatory modulation. Additionally, acceleration of ACLR graft maturation and incorporation was seen [21–23]. The authors present a case series detailing a novel surgical approach to improve the outcomes of allograft ACLR utilizing a resorbable type I collagen matrix (TenoMend™) (Exactech, Gainesville, FL) soaked in PRP (Accelerate® PRP) (Exactech, Gainesville FL) (Figure 1). The resorbable type I collagen matrix is bovine-derived and is both chemically and mechanically machined. These unique properties provide a porous scaffold that enables acceptance of the PRP while permitting cellular incorporation between the graft and bone interface. Furthermore, the wrap acts to shelter the intra-articular portion of the graft from the negative effects of plasmin, thus allowing for the valuable nutrients to survive in the intra-articular environment [24, 25].

## 2. Materials and Methods

Institutional Board Review (IRB) approval at the lead authors' institution was obtained for this retrospective evaluation. A retrospective chart review from 2014 to 2016 was performed looking specifically at patients who underwent quadruple-stranded hamstring allograft ACLR with a type I collagen matrix augment impregnated with PRP. Hybrid grafts and autografts were excluded. All outcomes were extrapolated from this chart review. Outcomes were defined as a failure if there was an ACL graft retear or instability requiring revision ACLR. A total of 14 ACLR were identified within this time frame that met the above criteria. All patients underwent the following arthroscopic technique.

Routine diagnostic arthroscopy is performed, and confirmation of the ACL tear is noted. Next, two nonirradiated semitendinosus hamstring allografts are selected from the cadaver bank and thawed in a warm Bacitracin-infused saline bath. The allografts are measured and cut to equal lengths. Each end of the allograft is then whip-stitched. Next, the allografts are doubled over an adjustable button suture loop (Rigidloop Adjustable™) (MITEK, Raynham, MA) and tensioned to 20 pounds of force on a standard prep board for 20 minutes.

Whole blood (60 cc) is obtained from the patient at the time of surgery in a sterile fashion from the upper extremity. The blood is processed and separated using a gentle centrifugal technique (Accelerate® Autologous Platelet Concentrating System) (Exactech, Gainesville, FL). The PRP, or buffy coat layer, is collected at 6-10 ml of solution. The platelet-poor plasma (PPP) is also collected (10 cc) for later use in the procedure.

**Figure 2:**
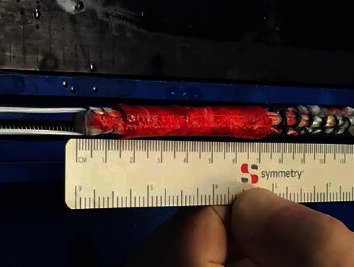
The soft tissue graft is tensioned on a standard prep board. The PRP-soaked TenoMend™ is sutured around the graft, ensuring coverage of the graft contained within the bone tunnels as well as the joint.

The collagen matrix is then cut to fit the semitendinosus graft and soaked in the PRP bath for a period of 5 minutes. Following this, the collagen wrap is placed around the hamstring allograft and sutured into place via a combination of 3-0 Vicryl interrupted and locking sutures (Figure 2). It should be noted that the collagen matrix is machined to come precurled, facilitating the ease of placement around the allograft tissue.

ACL tunnel preparation is then carried out in standard arthroscopic fashion via independent tunnel drilling consistent with an “anatomic” graft placement. The graft diameter had previously been sized at the conclusion of tensioning and prior to the application of the collagen graft. Typically, line-to-line drilling is undertaken; however, in instances where the graft is extremely snug in the sizer, the lead author elects to upsize by 0.5 mm for the tunnel drilling. On the tibial side, care is taken to preserve as much of the native ACL footprint as possible, allowing cellular migration from the native tissue across the graft and potentially leading to quicker ligamentization.

**Figure 3:**
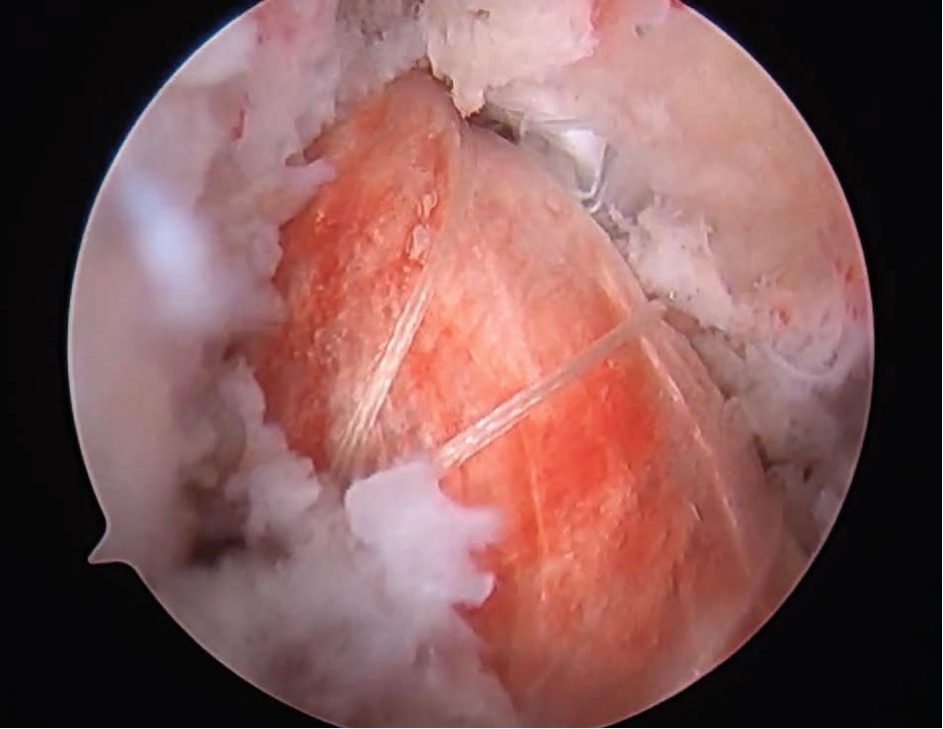
The graft has been secured into the femoral tunnel. The PRP-soaked collagen wrap maintains the biologic agent within the aqueous environment.

**Figure 4:**
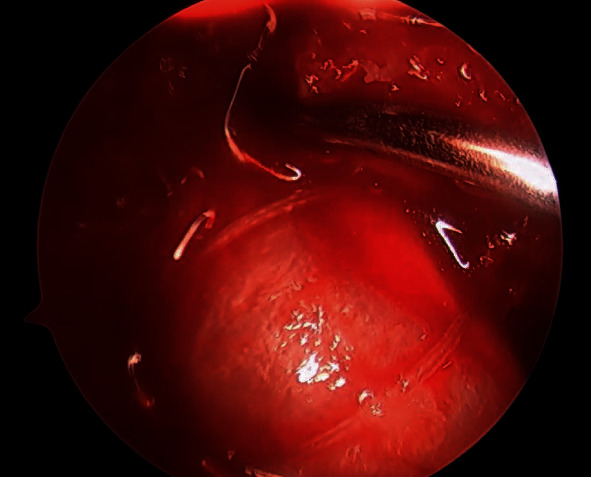
Under a dry environment, the remaining PRP is injected into the bone tunnels as well as within the collagen wrap.

Upon completion of the tunnel preparation, the graft is carefully shuttled up through the tibial and femoral tunnels, and the adjustable button is flipped onto the lateral femoral cortex (Figure 3). The graft is secured into the tibial canal utilizing a screw and sheath technique within the four limbs of the graft under 20 pounds of manual tension. After checking the isometry and tension of the graft, attention is turned to the remaining PRP. Under a “dry scope technique,” 3-4 ml of PRP is injected between the allograft tendons and the collagen wrap utilizing an 18-gauge spinal needle (Figure 4). The remaining PRP is then injected into the femoral and tibial tunnels. Finally, the previously harvested PPP is injected into the joint after the portals are closed. A standard soft tissue ACLR postoperative rehabilitation protocol is followed upon discharge.

## 3. Results

14 patients were identified to have undergone quadruple-stranded nonirradiated hamstring allograft ALCRs with a type I collagen wrap augmentation. The average age of the patients who underwent the procedure was 33 years of age (range 24-44). 0 out of 14 patients had reported rerupture at a minimum of 24 months postprocedure. None of the patients required further surgical intervention for instability. None of the patients had reported adverse events related to the surgical procedure. 1 of the 14 patients underwent repeat arthroscopy approximately 13 months postprocedure for a failed medial meniscus repair. The ACLR was noted to be intact at that time with signs of ligamentization.

## 4. Discussion

ACLR failure rates are a troubling problem for orthopedic surgeons, particularly in a younger or more athletic population. There is a disparity in the literature regarding the rate of ACLR failure rates [4, 6–12, 17, 19]. Contributing to the wide range of reported failure rates includes variation in graft choice, surgical technique, patient demographics, and the use of biologics as an augment. While each factor can have a certain measure of control placed upon them, it is nevertheless implicit upon the surgeon to explore all options that can optimize patient outcomes.

Several articles have discussed the risk of ACLR failure associated with allograft tissue [6–19]. A significant increase in ACLR failure has been shown in young patients who received allograft tissue in their reconstruction procedures [10–13, 19]. Kaeding et al., in a review of the data from the Multicenter Orthopaedic Outcomes Network (MOON) trials, found a four times higher ACLR graft failure rate when using allograft versus autograft in the population of 10–19 years. A meta-analysis of levels 2 and 3 studies, comparing primary patellar tendon autograft and allograft ACLRs, found approximately five times higher odds of graft rupture for patients who received allograft reconstructions [11]. Similarly, a meta-analysis of 20 studies by Prodromos et al. reported a 5% failure rate in autografts compared with a 14% failure rate in allografts (*P* < 0.01) [12]. Finally, a recent meta-analysis by Ellis et al. found allograft failure rates of 25.5% compared to 8.5% for bone-patella-bone autografts [13].

Delay in biologic graft incorporation has been of primary concern when considering allograft failures [19–21]. Rappé et al. found a 33% failure rate when using irradiated Achilles tendon allografts to a 2.4% failure rate when using nonirradiated Achilles tendon allografts [18]. The role of irradiation may weaken the integrity of the allograft tendon, predisposing it to tear in a highly active population. The advent of nonirradiated allografts has allowed for a decrease in the allograft failure rates; however, these numbers still exceed that of autograft ACLR outcomes [18]. Nonetheless, some surgeons or patients favor the use of allograft due to ease of procedure, availability without delay, decreased donor site morbidity, and decreased surgical time. Based upon this, the authors theorized that the addition of a porous type I collagen scaffold reconstituted with PRP could decrease the risk of failure by increasing the likelihood of biologic graft incorporation.

The concept of utilizing biologics as an augment for ACLR has gained popularity in recent years [19–23]. Fleissner et al. reported on 143 patients who underwent ACLR with autograft augmented with type I collagen matrix hydrated with PRP. In their series, the failure rate was 5% [19]. Of note, there was a 92% return to previous activity reported with return to play allowed, on average, at 22 weeks postprocedure. By way of comparison, Arden et al. noted in their ACLR group a return to preinjury sports participation of only 60% [26]. A second look arthroscopy on one ACLR for a separate injury demonstrated graft incorporation with neovascularization and ligamentization at 7 months postprocedure. Furthermore, Weiler et al. demonstrated that the adjunct of PRP on an ACLR can lead to advanced graft maturation and increased tensile strength [27]. Fleming et al. also found this to be true in their porcine model, further noting that biologic augmentation resulted in decreased ligament laxity [28].

The concept of utilizing type I collagen matrix, hydrated with a PRP clot to block plasmin from the healing environment, is a novel approach to a known hostile environment within the intra-articular knee joint. Murray et al. has done extensive work through their Bridge-Enhanced Anterior Cruciate Ligament Repair (BEAR) collagen scaffold research [29, 30]. The BEAR scaffold is applied to primary ACL repairs as opposed to ACLR. Nevertheless, one of the functions of the BEAR scaffold is to allow for and promote the formation of a protective fibrin clot between the native ACL and the remaining anatomic structures of the ACL within the knee joint. These authors note that one possible failure of previous work done on native ACL tear from healing is due to the failure of fibrin clot formation, which functions as a provisional scaffold within the ACL wound site. This deficiency is likely secondary to the ACL's location within the synovial cavity [2, 24, 25]. To circumvent this problem, collagen-platelet composites (CPCs) have been used as a substitute scaffolding material [25]. This scaffold simulates a fibrin clot, creating an environment conducive to healing within the gap between the torn ends of the ACL. Primary ACL repair is of particular interest because this treatment would avoid the morbidities associated with tissue harvest or allograft use, it would potentially retain the ligament insertion sites, and it could better preserve the proprioceptive nerves of the ACL.

This 14 patient case series on patients who underwent ACLR with a type I collagen augment impregnated with PRP demonstrated positive outcomes at of minimum 2 years postprocedure evaluation. The authors realize limitations exist. First, a small sample size is reported, and as such, this should be considered in the context of a case series. A second limitation is the lack of standardized laxity testing, such as KT-1000. Per standard postoperative protocol, the lead author does not routinely have this test performed on patients; however, chart review notes “negative Lachman” test at final follow-up on each of the 14 patients. Furthermore, there was the absence of subjective knee instability at the final follow-up. Anecdotally, the authors feel that there was less knee laxity in the patients who underwent the allograft ACLR with augmentation versus those who did not.

## 5. Conclusion

Improving patient outcomes in ACLR is a continuous journey for orthopedic surgeons. Many variables require contemplation by the treating surgeon when performing an ACLR. In situations where allograft tissue is to be used, steps should be taken to aid in the process of biologic graft incorporation. The authors feel that they present a compelling case series offering a novel approach to allograft ACLR utilizing a type I collagen wrap impregnated with PRP. Further investigation is warranted into this technique including a larger sample size, KT-1000 testing, and potentially tissue biopsy to evaluate for tissue viability.

## Data Availability

The retrospective outcomes data used to support the findings of this study are included within the article.

## References

[1] Gordon M. D., Steiner M. E., Garrick J. G. (2004). Anterior cruciate ligament injuries. *Orthopaedic Knowledge Update Sports Medicine III*.

[2] Musahl V., Karlsson J. (2019). Anterior cruciate ligament tear. *The New England Journal of Medicine*.

[3] Herzog M. M., Marshall S. W., Lund J. L., Pate V., Spang J. T. (2017). Cost of outpatient arthroscopic anterior cruciate ligament reconstruction among commercially insured patients in the United States, 2005-2013. *Orthopaedic Journal of Sports Medicine*.

[4] Noyes F. R., Barber-Westin S. D. (2001). Revision anterior cruciate surgery with use of bone-patellar tendon-bone autogenous grafts. *The Journal of Bone and Joint Surgery. American Volume*.

[5] Bach B. R., Provencher M. T. (2010). *ACL Surgery: How to Get it Right the First Time and What to Do if it Fails*.

[6] Morgan J. A., Dahm D., Levy B., Stuart M. J., and the MARS Study Group (2012). Femoral tunnel malposition in ACL revision reconstruction. *The Journal of Knee Surgery*.

[7] Di Benedetto P., Di Benedetto E., Fiocchi A., Beltrame A., Causero A. (2016). Causes of failure of anterior cruciate ligament reconstruction and revision surgical strategies. *Knee Surgery & Related Research*.

[8] Spindler K. P., Kuhn J. E., Freedman K. B., Matthews C. E., Dittus R. S., Harrell F. E. (2004). Anterior cruciate ligament reconstruction autograft choice: bone-tendon-bone versus hamstring: does it really matter? A systematic review. *The American Journal of Sports Medicine*.

[9] Samitier G., Marcano A. I., Alentorn-Geli E., Cugat R., Farmer K. W., Moser M. W. (2015). Failure of anterior cruciate ligament reconstruction. *Archives of bone and joint surgery*.

[10] Kaeding C. C., Aros B., Pedroza A. (2011). Allograft versus autograft anterior cruciate ligament reconstruction: predictors of failure from a MOON prospective longitudinal cohort. *Sports Health: A Multidisciplinary Approach*.

[11] Krych A. J., Jackson J. D., Hoskin T. L., Dahm D. L. (2008). A meta-analysis of patellar tendon autograft versus patellar tendon allograft in anterior cruciate ligament reconstruction. *Arthroscopy: The Journal of Arthroscopic & Related Surgery*.

[12] Prodromos C., Joyce B., Shi K. (2007). A meta-analysis of stability of autografts compared to allografts after anterior cruciate ligament reconstruction. *Knee Surgery, Sports Traumatology, Arthroscopy*.

[13] Cruz A. I., Beck J. J., Ellington M. D. (2020). Failure rates of autograft and allograft ACL reconstruction in patients 19 years of age and younger. *JBJS Open Access*.

[14] Ménétrey J., Duthon V. B., Laumonier T., Fritschy D. (2008). “Biological failure” of the anterior cruciate ligament graft. *Knee Surgery, Sports Traumatology, Arthroscopy*.

[15] Carey J. L., Dunn W. R., Dahm D. L., Zeger S. L., Spindler K. P. (2009). A systematic review of anterior cruciate ligament reconstruction with autograft compared with allograft. *The Journal of Bone and Joint Surgery. American Volume*.

[16] Scheffler S. U., Schmidt T., Gangéy I., Dustmann M., Unterhauser F., Weiler A. (2008). Fresh-frozen free-tendon allografts versus autografts in anterior cruciate ligament reconstruction: delayed remodeling and inferior mechanical function during long-term healing in sheep. *Arthroscopy*.

[17] Poehling G. G., Curl W. W., Lee C. A. (2005). Analysis of outcomes of anterior cruciate ligament repair with 5-year follow-up: allograft versus autograft. *Arthroscopy*.

[18] Rappé M., Horodyski M., Meister K., Indelicato P. A. (2007). Nonirradiated versus irradiated Achilles allograft: in vivo failure comparison. *The American Journal of Sports Medicine*.

[19] Berdis A. S., Veale K., Fleissner P. R. (2019). Outcomes of anterior cruciate ligament reconstruction using biologic augmentation in patients 21 years of age and younger. *Arthroscopy: The Journal of Arthroscopic & Related Surgery*.

[20] Looney A. M., Leider J. D., Horn A. R., Bodendorfer B. M. (2020). Bioaugmentation in the surgical treatment of anterior cruciate ligament injuries: a review of current concepts and emerging techniques. *SAGE Open Medicine*.

[21] Andriolo L., di Matteo B., Kon E., Filardo G., Venieri G., Marcacci M. (2015). PRP augmentation for ACL reconstruction. *BioMed Research International*.

[22] Vavken P., Sadoghi P., Murray M. M. (2011). The effect of platelet concentrates on graft maturation and graft-bone interface healing in anterior cruciate ligament reconstruction in human patients: a systematic review of controlled trials. *Arthroscopy: The Journal of Arthroscopic & Related Surgery*.

[23] Vogrin M., Rupreht M., Dinevski D. (2010). Effects of a platelet gel on early graft revascularization after anterior cruciate ligament reconstruction: a prospective, randomized, double-blind, clinical trial. *European Surgical Research*.

[24] Günther A., Markart P., Kalinowski M., Ruppert C., Grimminger F., Seeger W. (1999). Cleavage of surfactant-incorporating fibrin by different fibrinolytic agents. Kinetics of lysis and rescue of surface activity. *American Journal of Respiratory Cell and Molecular Biology*.

[25] Kroon M. E., van Schie M. L., van der Vecht B., van Hinsbergh V. W., Koolwijk P. (2002). Collagen type 1 retards tube formation by human microvascular endothelial cells in a fibrin matrix. *Angiogenesis*.

[26] Ardern C. L., Webster K. E., Taylor N. F., Feller J. A. (2011). Return to sport following anterior cruciate ligament reconstruction surgery: a systematic review and meta-analysis of the state of play. *British Journal of Sports Medicine*.

[27] Weiler A., Förster C., Hunt P. (2017). The influence of locally applied platelet-derived growth factor-BB on free tendon graft remodeling after anterior cruciate ligament reconstruction. *The American Journal of Sports Medicine*.

[28] Fleming B. C., Spindler K. P., Palmer M. P., Magarian E. M., Murray M. M. (2009). Collagen-platelet composites improve the biomechanical properties of healing anterior cruciate ligament grafts in a porcine model. *The American Journal of Sports Medicine*.

[29] Mastrangelo A. N., Vavken P., Fleming B. C., Harrison S. L., Murray M. M. (2011). Reduced platelet concentration does not harm PRP effectiveness for ACL repair in a porcine in vivo model. *Journal of Orthopaedic Research*.

[30] Murray M. M., Kalish L. A., Fleming B. C. (2019). Bridge-Enhanced Anterior Cruciate Ligament Repair: Two-Year Results of a First-in-Human Study. *Orthopaedic journal of sports medicine*.

